# Similar recovery time of microbial functions from fungicide stress across biogeographical regions

**DOI:** 10.1038/s41598-018-35397-1

**Published:** 2018-11-19

**Authors:** Verena C. Schreiner, Alexander Feckler, Diego Fernández, Katharina Frisch, Katherine Muñoz, Eduard Szöcs, Jochen P. Zubrod, Mirco Bundschuh, Jes J. Rasmussen, Ben J. Kefford, Josepha Axelsen, Nina Cedergreen, Ralf B. Schäfer

**Affiliations:** 1 Institute for Environmental Sciences, University Koblenz-Landau, Fortstraße 7, 76829 Landau in der Pfalz, Germany; 2 Department of Aquatic Sciences and Assessment, Swedish University of Agricultural Sciences, Box 7050, 75007 Uppsala, Sweden; 3 Present Address: BASF SE, 67056 Ludwigshafen am Rhein, Germany; 4 Department of Bioscience, Aarhus University, Vejlsøvej 25, 8600 Silkeborg, Denmark; 5 Institute for Applied Ecology, University of Canberra, ACT, 2601 Canberra, Australia; 6 Department of Plant and Environmental Sciences, University of Copenhagen, Thorvaldsensvej 40, 1871 Frederiksberg, Denmark

## Abstract

Determining whether the structural and functional stress responses of communities are similar across space and time is paramount for forecasting and extrapolating the consequences of anthropogenic pressures on ecosystems and their services. Stream ecosystems are under high anthropogenic pressure; however, studies have only examined the response of stream communities across large scales over multiple generations. We studied the responses of leaf-associated microbial communities in streams within three European biogeographical regions to chemical stress in a microcosm experiment with multiple cycles of fungicide pollution and resource colonisation. Fungal community composition and the ecosystem function leaf decomposition were measured as response variables. Microbial leaf decomposition showed similar recovery times under environmental levels of fungicide exposure across regions. Initially, the decomposition declined (between 19 and 53%) under fungicide stress and recovered to control levels during the third cycle of pollution and colonisation. Although community composition and its stress response varied between regions, this suggests similar functional community adaptation towards fungicide stress over time. Genetic, epigenetic and physiological adaptations, as well as species turnover, may have contributed to community adaptation but further studies are required to determine if and to which extent these mechanisms are operating. Overall, our findings provide the first evidence of a similar functional response of microbial leaf decomposition to chemical stress across space and time.

## Introduction

Human activities are altering ecosystems globally at an unprecedented magnitude. In this context, streams are among the ecosystems with the highest risk of biodiversity loss, and such losses may hamper the perpetuation of crucial ecosystem services. Major anthropogenic stressors for stream ecosystems include habitat degradation, climate change, nutrient enrichment, and chemical pollution (e.g., pesticides)^1^. Several of these stressors, such as climate change and nutrient enrichment, are close to or are already exceeding their planetary boundaries (i.e. the stressor-related global biophysical thresholds)^2^. Crossing these boundaries could result in irreversible systemic state shifts with adverse consequences for human societies^3^. However, a planetary boundary for man-made chemical pollution has not yet been quantified. Although the physico-chemical properties of certain pollutants allow for their long-range transport and occurrence on a global scale^4^, chemical pollution predominantly affects local processes^5^. Setting thresholds, such as planetary boundaries, as well as to reliably extrapolate results across regions would require a similar local stress response^6,7^.

Few studies have examined ecological stress responses in stream ecosystems across large biogeographical scales^8–13^. Similar responses from regional to global scales have been observed in assemblages of macroinvertebrate and fish communities to urbanisation^8^, pesticides^11^, and hydromorphological disturbances^9^. For macroinvertebrate-mediated leaf decomposition, which is an important energy-providing function in stream ecosystems^14^, similar responses towards nutrient enrichment were found on a European scale^10^, whereas the impacts of temperature changes at European^13^ and worldwide scales^12^ were partially contradictory. Moreover, it remains largely unexplored if stress responses across regions show a similar temporal pattern. This limits our ability to identify general mechanisms underlying community responses such as evolutionary (e.g. genetic adaptation) and ecological processes (e.g. competition) as well as eco-evolutionary feedbacks^15^. Microbial communities represent an ideal organism group to study temporal and spatial stress response patterns because of their high reproduction rates and rapid adaptation to stress^16^.

We examined the functional (i.e., leaf decomposition) and structural (i.e., community dynamics) stress responses of microbial communities in different European biogeographical regions over multiple cycles of pollution and resource colonisation. We used a model system consisting of leaf-associated microbial decomposer communities and a fungicide mixture as a stressor. One part of these microbial communities is the polyphyletic fungal group of aquatic hyphomycetes that are crucial for leaf decomposition in streams^17^. Several representatives of this group are cosmopolitan^18^, thereby allowing for comparisons of taxa-specific spatial responses. Fungicides were chosen as the stressor because they are designed to suppress fungal pathogens and are known to affect non-target fungi, such as aquatic hyphomycetes^19^. In addition, fungicides are transported into surface waters during or after their application in catchments^20^. The involvement of multiple hyphomycete colonisation and decomposition cycles enabled studying potential microbial community acclimatisation and adaptation to fungicide stress. The experiment was performed with communities from three biogeographical regions (the Central Plains, Denmark; the Western Highlands, Germany; and the Fenno-Scandian Shield, Sweden) to detect if functional as well as structural stress responses caused by fungicides are similar across space.

Using this experimental design, we found an initial reduction in leaf decomposition by fungicide exposure, followed by recovery within a similar time period. This similar recovery period suggests functional adaptation towards fungicide pollution over the course of the experiment. The structural responses in terms of shifts in the aquatic hyphomycete community composition, however, varied across the biogeographical regions, with the number of colonisation and decomposition cycles, fungicide exposure or the interaction of these factors acting as the main drivers of the effects.

## Results

### General experimental design

**Figure 1:**
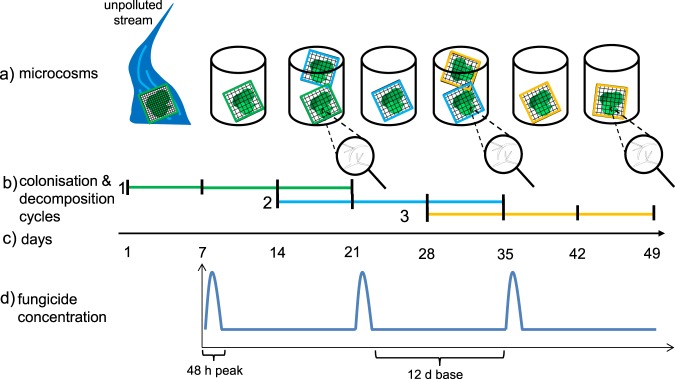
Schematic illustration of the experimental design. Coloured lines in the middle of the figure indicate leaf sets, which correspond to the three colonisation and decomposition cycles (**b**). The first leaf set (green) was colonised in unpolluted streams for 1 week and subsequently used in the experiment. Leaf sets 2 and 3 (blue and orange, respectively) were colonised in the microcosms by the microbial community from previous leaf sets for 1 week (overlapping bars) (**a,c**). At the end of each cycle, the decomposed leaf mass was quantified, and the aquatic hyphomycete community composition was determined based on fungal spore production. The fungicide exposure pattern (three 48-hour peaks, each followed by 12-day baseline exposures) is depicted in the lower panel (d).

The experiments were conducted in three biogeographical regions (Central Plains, Silkeborg, Denmark; Western Highlands, Landau/Pfalz, Germany; and Fenno-Scandian Shield, Uppsala, Sweden)^21^ over seven weeks following the same protocol (for details, see the Methods section). Briefly, three consecutive sets of leaf material were used that corresponded to three microbial colonisation and decomposition cycles (Fig. 1a,b). While the first leaf set was colonised by microbial communities in unpolluted streams, the other two leaf sets were colonised in microcosm systems by the leaf-associated microbial community present on the leaves of the previous cycle. With this setup, potential adaptations of the whole aquatic hyphomycete communities under fungicide stress were captured over the three consecutive cycles. However, adaptation on the community level integrates various factors including: genetic, epigenetic and physiological (e.g. phenotypic plasticity) adaptations as well as species turnover, of which we only measured the latter indirectly through sporulation.

Vegetative growth and reproduction both contribute to the community dynamics in aquatic hyphomycetes, where reproduction is typically triggered through resource (leaf) availability, which can be periodical^22^. Our setup allowed us to track the implications on reproduction because the colonisation of new resources through sporulation is usually more sensitive towards stress than vegetative growth^23^. Several studies using both sporulation and molecular methods found that the strongest dynamic in community composition and sporulation of aquatic hyphomycetes typically occurs within the first two to three weeks after the initial colonisation^17,22^. Therefore, we set the duration of each cycle to three weeks.

The microcosms were subjected to two treatments: a control treatment and a fungicide treatment. The latter consisted of 48-hour peak exposures followed by 12-day base exposures with fungicide concentrations of 10% of the peak (Fig. 1d). This exposure pattern mimicked the higher levels of pesticide toxicity identified in a meta-analysis of global pesticide levels^11^ and the subsequent baseline exposure resulting from drainage within catchments^24^ (information on the fungicide mixture is presented in Supplementary Table 1).

### Functional responses

**Figure 2:**
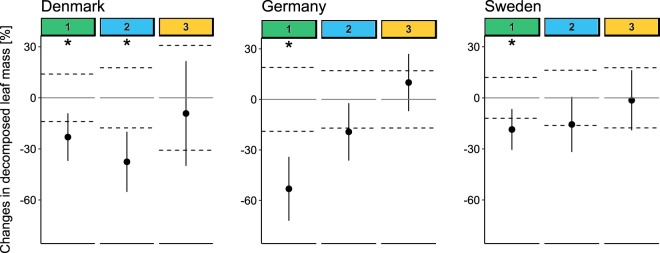
Mean percentage change in decomposed leaf mass. Changes in the decomposed leaf mass under fungicide stress compared with the respective controls (in %, with 95% confidence intervals; solid horizontal lines represent the controls, and dashed lines indicate the corresponding 95% confidence intervals) for the different cycles (numbers on top; colour code refers to Fig. 1; Denmark and Germany n = 7 and Sweden n = 6). Significant differences at α = 0.05 between the controls and the fungicide treatments (t-test; Supplementary Table 2) are depicted with asterisks.

Leaf decomposition showed a different temporal stress response across biogeographical regions (interaction of factors “cycle” and “region”: likelihood ratio = 19.2, p < 0.001). This was mainly due to different effect sizes related to the initial reduction in leaf decomposition by fungicide exposure. The effect size for Germany in the first cycle was twofold higher (approximately 55%) than for Denmark and Sweden (approximately 25 and 20%, respectively), and the reduction was significant in all regions (Fig. 2; Supplementary Table 2). This effect attenuated during the subsequent cycles and leaf decomposition approached control levels during the third cycle in all regions (Fig. 2; Supplementary Table 2). Thus, despite different effect sizes, the overall effect pattern and recovery time were similar across regions. Indeed, the functional effect pattern and recovery time over the cycles matched with additional data from the South Eastern Highlands (Australia), which highlights the potential transferability of the functional responses to other regions (Supplementary Table 4 and Supplementary Fig. 1).

### Structural responses

Cosmopolitan hyphomycete taxa occurring in all three experiments showed contrasting responses to fungicide stress and the cycles in terms of spore production (type II analysis of variance, ANOVA). For example, the spore production of *Articulospora tetracladia* (Ingold) decreased with fungicide exposure over the cycles in Denmark but not in Germany and Sweden. The same taxon also responded to cycles in the absence of fungicides in Sweden but not in Denmark and Germany. The sporulation of *Tetrachaetum elegans* (Ingold) decreased over the cycles in the absence and presence of fungicides in Germany but not in Denmark and generally sporulated at very low densities in Sweden (Supplementary Table 3).

**Figure 3:**
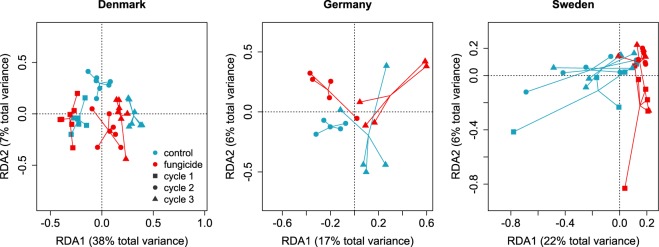
Hyphomycete community composition across cycles and treatments. Each point within the RDAs represents the community of one replicate. Replicates of one treatment are connected through lines (Denmark n = 7, Germany n = 5 and Sweden n = 6). Because of technical difficulties, data are not available for the hyphomycete community from the first cycle in Germany.

Redundancy analyses (RDAs) revealed different drivers of structural responses among the biogeographical regions (Fig. 3). In Denmark, the interaction between cycles with fungicide exposure was significant and explained approximately 9% of the variation. Although care should be taken when interpreting the main effects in the presence of interactions, the cycle seemed to have the highest individual influence on the community composition (explained variance: 41%; Table 1), which was also the only significant variable for the community composition in Germany that explained 17% of the variance. In Sweden, however, fungicide exposure was the main driver of the community changes (explained variance: 22%), and only a minor impact of the cycle (8%) and a non-significant interaction between the cycle and fungicide exposure were observed (Table 1).

**Table 1:** Explained variance and p-values of the RDA variables tested by type III ANOVAs, separated by biogeographical region and explanatory variables. Bold p-values indicate statistical significance.

Region	Explanatory variable	df	t	p-value	Explained variance (%)
Denmark	Fungicide	1	1.2	0.263	2.9
	Cycle	2	13.7	**0.001**	41.3
	Fungicide × Cycle	2	3.6	**0.002**	9.3
Germany	Fungicide	1	1.2	0.299	6.3
	Cycle	1	3.6	**0.001**	16.5
	Fungicide × Cycle	1	1.1	0.365	5.1
Sweden	Fungicide	1	9.1	**0.001**	21.6
	Cycle	2	1.4	0.224	8.0
	Fungicide × Cycle	2	0.4	0.917	1.5

## Discussion

We found similar effect patterns and recovery time periods of leaf decomposition from fungicide exposure across biogeographical regions. More specifically, leaf decomposition declined during the first cycle, but this effect was attenuated during the following two cycles (Fig. 2). The initial response is typical for leaf-associated microbes inhabiting unpolluted systems when exposed towards organic fungicides^25^ that predominantly inhibit the biosynthesis of ergosterol, RNA and proteins^26^ and consequently affect the growth or activity of aquatic hyphomycetes by compromising their leaf decomposition ability. The similar time spans of recovering leaf decomposition during the following cycles in all regions (Fig. 2) suggest that the microbial communities, which originated from unpolluted streams, adapted to fungicide stress and ultimately recovered their functional performance irrespective of slight differences in the applied fungicide mixture (Supplementary Table 1). This can be explained by the concept of pollution-induced community tolerance (PICT), which assumes that communities increase their overall tolerance as a result of genetic, epigenetic or physiological (e.g. phenotypic plasticity) adaptations and/or species turnover and thus functional responses are not, or minimally, affected under stress^27^. Although our data suggest that a PICT was developed during the present study, identification of the dominant underlying mechanisms (e.g. genetic and species turnover) resulting in community adaptation requires future studies^28,29^. The functional pattern was similar despite different fungal communities in the test systems, and relatively stable decomposition patterns without fungicides across the cycles in Germany, whereas slight and stronger declines in decomposition over time were observed in Sweden and Denmark, respectively (Supplementary Fig. 2).

The similarity in the pattern of the functional response and its recovery time span was not reflected in the structural responses, neither by means of the cosmopolitan occurring fungal species nor by taxa richness of the aquatic hyphomycete communities across biogeographical regions. Chemical stress can act as a strong selection pressure^30^ and an environmental filter, through which sensitive taxa are replaced by tolerant competitors. If environmental filtering (here fungicide stress) is the only driver for the community structure, then cosmopolitan aquatic hyphomycete taxa should show a similar response across spatial scales^31^. However, the cosmopolitan hyphomycete taxa occurring in communities from all biogeographical regions (Supplementary Table 3) responded differently to fungicide exposure and the cycles. This observation supports Cadotte and Tucker^15^, who stressed the importance of other mechanisms, such as ecological processes (e.g. competition and predation) and the interaction of biotic processes with environmental filtering, which may be the main drivers of responses at the community level.

The taxa richness of the hyphomycete communities declined in all biogeographical regions. This, as well as shifts in the composition of the hyphomycete communities, exhibited partially different drivers among the regions (Fig. 3; Table 1; Supplementary Table 5). Sporulation within all three biogeographical regions was mostly driven by one or two hyphomycete taxa per replicate, which contributed between 50 and 100% of the total sporulation. When assessing the taxon richness based on sporulation, Denmark and Sweden showed comparable tendencies. The sporulating aquatic hyphomycete taxa decreased by up to 50% over the cycles in both the fungicide treatment and the control (Supplementary Table 5, Supplementary Fig. 3). This decrease was driven by the elimination of hyphomycete taxa with low sporulation rates, which seems common in microcosms^32^. Taxa with lower relative sporulation rates were potentially outcompeted by those with higher sporulation rates when colonising the leaves of the next cycle^33^. However, the sporulation peaks vary based on the taxon and the environment^17,34^, and under field conditions, low sporulating taxa from the first cycle might sporulate more strongly subsequently. Notwithstanding, non-sporulation does not translate to absence, and the taxa may still have been present and contributed to decomposition during all cycles^35^. The pattern of decreases in sporulating taxa may also apply to the communities from Germany, although the lack of data from the first colonisation cycle prohibits a definite interpretation (see the Methods section). Nevertheless, the minor importance of fungicide exposure for community dynamics in Denmark and Germany was reflected in a relatively similar taxa richness and composition across the treatments and cycles (Fig. 3; Supplementary Tables 3 and 5, Supplementary Fig. 2).

This similarity suggests that the taxa thriving under experimental conditions may also exhibited a higher tolerance towards fungicides and may have driven decomposition. Similarly, the Swedish communities consisted of the same taxa irrespective of the fungicide treatment (Supplementary Table 3). Nevertheless, the sporulation of fungal taxa was reduced by fungicides, suggesting fungicide stress was the main driver for this structural response (Fig. 3, Table 1).

The difference in the effect sizes of leaf decomposition between Sweden and Germany during the first cycle and its faster attenuation in Sweden compared to Denmark (Fig. 2) might be explained by the higher dissolved organic carbon content of the stream water used as a test medium in Sweden (48 mg/L) compared with Germany (<1 mg/L) and Denmark (1.3 mg/L)^36^. The associated enzymatic inventory of certain aquatic hyphomycetes allows them to degrade and utilise organic compounds (including xenobiotics) as an alternative source of nutrients^37^. Energetic investments in detoxification processes and the supply of additional nutrients could, to some extent, have reduced the fungicide stress experienced by the whole community and facilitated higher leaf decomposition.

Although general conclusions derived from communities of only one single stream per biogeographical region and stressor concentration are considered speculative, our findings represent the first evidence of a similar functional response of microbial leaf decomposition to chemical stress across space and time. Such knowledge on similar stressor responses could help to forecast future stress responses, which is important to inform managers of ecosystems aiming to reduce human-induced effects through mitigation measures^6,7^, but also to model ecosystem response towards future stress. Additionally, comparing stress responses across large biogeographical scales should inform efforts to establish planetary boundaries for chemical pollution. Finally, our study implies that the future risk assessment of chemicals needs to focus on both, ecosystem function and community composition, as their response patterns can differ.

Future studies should scrutinise the response to multiple concentrations levels and mechanisms of community adaptation. While microbial communities have been used as model systems for eco-evolutionary processes and stress adaptations, the translation of the results to higher trophic levels that drive important stream ecosystem functions (e.g., insects or vertebrates) remains open. Although community adaptations of higher trophic levels to stress are likely to occur over longer time periods, because of longer generation times, the steadily increasing stress load under climate change and increasing human populations may exceed the time frame required for adaption, exacerbating functional losses^38^. Moreover, although microbial systems are informative regarding the potential occurrence of similar responses, higher-level studies linking short-term, eco-genomic approaches with ecological models are required. Such studies may ultimately help to determine the effect thresholds for ecosystem management and the planetary boundaries for chemical stressors.

## Methods

### Fungicide selection and application scenarios

A mixture of three to four fungicides with distinct modes of toxic action was used during the experiment (inhibitors of ergosterol, RNA and protein synthesis^26^; see Supplementary Table 1). Microcosms were spiked individually using fungicide mixture solutions shortly before test initiation and after media renewal between the peak and baseline exposure scenario (see below) to achieve the nominal concentrations. Each of the fungicides contributed equally to the toxic potential of the mixtures based on the effective concentration that reduces the growth of test organisms (hyphomycetes when data were available; otherwise, *Pseudokirchneriella subcapitata;* Supplementary Table 1). The fungicides were applied as short-term episodic peaks (48 h) at levels representing the upper end of pesticide toxicity in the field identified in a meta-analysis^39^. These peaks were interspersed by base exposures at 10-fold lower concentrations (12 d; see Fig. 1; for more details see Supplementary Information)^40^. A fungicide-free control treatment was run in parallel to this fungicide treatment. The test medium (pre-filtered stream water) in the microcosms together with the respective fungicide concentrations was renewed after each peak exposure until 7 d after base exposure to ensure constant water quality and exposure to the fungicides. Liquid chromatography high-resolution mass spectrometry was used to verify the fungicide concentrations in the respective microcosm experiments (see Supplementary Tables 6 and 7).

### Leaf decomposition

Senescent but undecomposed leaves (*Alnus glutinosa* (L.) Gaertn.) were collected shortly before leaf fall and stored at −20 °C (for coordinates, see Supplementary Table 9). The soluble leaf components were leached from the leaves in aerated ultrapure water for 48 h before the experiment. This leaching can result in a mass loss of up to 30%^41^. Next, the leaves were dried to constant weight at 60 °C, and sets of leaf material were prepared (per replicate: 4 g (Germany; microcosm volume: 4 L) or 2 g (Denmark and Sweden; microcosm volume: 1 L), all ±0.01 g) and packed into nylon fine-mesh leaf bags (mesh size: 2 mm). For the first colonisation and decomposition cycle, leaf bags (Denmark and Germany: n = 14, Sweden: n = 12) were submerged for 7 days in unpolluted streams for microbial colonisation upstream of any urban or agricultural influence (Fig. 1; for the coordinates, see Supplementary Table 10). Afterwards, the leaf bags were retrieved, and the leaf material was introduced into the microcosms after the removal of invertebrates and sediment under running tap water. The microcosms contained pre-filtered water from the respective stream sites (to reduce confounding effects on aquatic hyphomycetes caused by changes in water quality; the abiotic water parameters are shown in Supplementary Table 8) and were spiked with the fungicide mixtures of the respective concentrations. For cycles 2 and 3, the leaf bags were directly inserted into the microcosms (after re-soaking in ultrapure water to avoid floating) to allow for colonisation by the microbial assemblages from the previous cycle (Fig. 1c,d). The experiment was conducted under aeration in darkness and at a temperature as close as technically possible to the mean temperature of the stream where the leaves were colonised (Supplementary Table 8). At the end of each cycle (i.e., at days 21, 35, and 49; Fig. 1), the remaining leaf material was retrieved from the microcosms, leaf discs were cut for the aquatic hyphomycete analyses (see below), and the remaining leaf material was dried to constant weight at 60 °C and weighed to the nearest 0.01 g. The decomposed leaf mass (DLM) per degree day was calculated for each replicate as follows:

$$DLM=\frac{(\frac{{S}_{i}(0)-{S}_{i}(t)}{{S}_{i}(0)})\times 100}{\frac{({\sum }_{j}^{t}{\bar{T}}_{i}(j))}{{n}_{j}}}$$

where *S* is the leaf mass as a function of deployment time *t*; $\bar{T}$ is the mean temperature for a day *j* of each replicate *i*; *S*_*i*_*(0)* is the leaf mass at the start of each cycle, and *n*_*j*_ refers to the number of experiment days.

We conducted an additional experiment with the same setup in the South Eastern Highlands (Canberra, Australia)^42^ to assess the transferability of the data on the functional response obtained in Europe. In this additional experiment, only the decomposed mass of *Eucalyptus camaldulensis* (Dehnh.) leaves was quantified following the same test protocol (Fig. 1). Because of the absence of information on the hyphomycete community composition, we present these data solely in the Supplementary Information. Due to technical difficulties, data on the decomposed leaf mass of cycle 1 are missing.

### Aquatic hyphomycete community dynamics

We evaluated the aquatic hyphomycete community dynamics based on the number of produced fungal spores because sporulation should have a higher sensitivity to stress than vegetative growth^23^. To this end, five leaf discs ($\varnothing 1$ cm) were cut from randomly chosen leaves of each replicate and pooled as one sample (Denmark: n = 7, Germany: n = 5, Sweden: n = 6) at the end of each cycle (i.e., at days 21, 35, and 49; Fig. 1). To induce the sporulation of aquatic hyphomycetes, the leaf discs were submerged in water of the respective replicate and orbitally shaken at 120 rpm in darkness at the respective experimental temperature (Supplementary Table 8). After 72 h, conidia were prevented from agglomerating using 0.5% Tween80 and fixed in 2% formalin, and the samples were stored at 4 °C. Directly before identification, an aliquot of the conidia suspension was homogeneously filtered over a gridded membrane filter (0.45 µm), and retained conidia were stained with lactophenol cotton blue. At least 300 conidia were identified for each replicate (100–400x magnification) primarily using the key provided by Gulis *et al*.^43^. The number of counted conidia was normalised to the total filter surface, sample volume and dry weight of the respective set of leaf discs. Because of a malfunction of the orbital shaker, conidial identification was not possible for the first cycle in Germany.

### Data analysis

For the statistical analyses of the decomposed leaf mass, a linear mixed-effect model was fitted, where the response was the percentage difference of the fungicide treatment to the fungicide-free control across all biogeographical regions. This model included the factors “region” (categorical; Denmark, Germany, and Sweden) and “cycle” (categorical; 1, 2, and 3) and their interaction, with the replicates nested within the regions. Subsequently, we fitted separate models for the different biogeographical regions using the absolute decomposition values, to identify potentially contrasting drivers of changes in decomposition and simplify interpretation. The factors “cycle” and “fungicide exposure” (categorical; control and treated) and their interaction were included as explanatory variables in all these models. The factor cycle was used as a fixed factor, since the trajectory was of interest, and not used as random factor for temporally dependent data. The effects of these explanatory variables as well as their interactions on the decomposed leaf material were analysed by type II ANOVAs using F-tests. To distinguish significant differences between the fungicide treatment and the respective control, t-tests of each cycle and biogeographical region were run separately to focus on the differences within and not between the cycles. The p-values were adjusted for multiple testing using the multivariate t distribution according to Hothorn *et al*.^44^. Additionally, the percent change compared with the respective control treatment and the 95% confidence intervals are provided.

Differences in the aquatic hyphomycete community composition were analysed - separately for the different regions - using RDAs with Hellinger transformation to achieve standardisation and circumvent problems associated with the Euclidean distance for ecological data^45^. We run separate models because the communities differed vastly between the regions, which on the one hand reduces the power to detect responses of taxa occurring in single regions and on the other hand may mask potentially contrasting responses of the same taxa in different regions. The proportion of variance explained by the explanatory variables was first calculated by individual models for either the cycle or the fungicide treatment and then calculated using both variables together, including their interaction. The significance of the RDAs was checked using permutational type III ANOVAs^46^. Analyses of the changes in the occurrence of single hyphomycete taxa were analogous with the analyses of the remaining leaf mass. All statistical analyses and graphs were conducted in R (version 3.3.3)^47^ and supplemented by the required add-on packages. The term “significant(ly)” is exclusively used in the sense of “statistical significance” at a level of 0.05.

## Electronic supplementary material

Supplementary Information

## Data Availability

The datasets analysed during the current study are available in the GitHub repository https://github.com/rbslandau/schreiner_simstress.
