# Supplementary material for: Similar recovery time of microbial functions from fungicide stress across biogeographical regions
Source: Sci Rep. 2018 Nov 19;8:17021. doi: 10.1038/s41598-018-35397-1 (PMC6242862; doi:10.1038/s41598-018-35397-1)
Supplement: Supplementary file 1 — Supplementary Information [file 41598_2018_35397_MOESM1_ESM.pdf]

Supplementary information for the paper:

Similar recovery time of microbial functions from fungicide stress across biogeographical regions

Verena C. Schreiner, Alexander Feckler, Diego Fernández, Katharina Frisch, Katherine Muñoz, Eduard Szöcs, Jochen P. Zubrod, Mirco Bundschuh, Jes J. Rasmussen, Ben J. Kefford, Josepha Axelsen, Nina Cedergreen & Ralf B. Schäfer

Fungicide concentrations

A mixture of the fungicides metalaxyl, prothioconazole, pyrimethanil, and prochloraz was used as a model stressor during the experiments. These four fungicides have partially dissimilar modes of action and inhibit RNA synthesis in ribosomes (metalaxyl), methionine synthesis (pyrimethanil), or ergosterol synthesis (prothioconazole and prochloraz)[1]. The concentrations of the single compounds in the mixture were chosen to reach equal toxicity equivalents, which were calculated using the logarithmic sum of toxic units (sumTU):

$$sumTU = log\left(\sum_{i=1}^{n} \frac{c_i}{EC_{50_i}}\right)$$

where c_i is the concentration of the fungicide *i* and EC_{50i} is the concentration at which 50% of the test organisms of the reference species (Supplementary Table 1) were affected by exposure to fungicide *i*.

The concentrations displayed in Supplementary Table 1 are nominal peak concentrations and equal a sumTU of -1, whereas the base concentration was 10% of this value, i.e., sumTU of -2. Because of the technical difficulties regarding solubility, prochloraz was only applied during the first colonisation and decomposition cycle in Germany and was not applied in the Swedish experiment. Despite differences in the compounds used in the mixture, which consisted of three and four fungicides, the same modes of toxic action were implicated in all exposures because prothioconazole and prochloraz both inhibit ergosterol synthesis[1]. When discarding prochloraz from the mixture, the concentrations of the other pesticides were adjusted to sustain the chosen stressor intensity (see Supplementary Table 1). We suggest that this difference was irrelevant for the observed patterns, which is supported by the fact that i) functional effects were equal or less pronounced in Denmark and ii) structural responses were similar to those in Germany. If the inclusion of prochloraz had a major

influence, Denmark should have displayed a deviating pattern from Germany and Sweden, which was not the case.

Supplementary Table 1: EC_{50} values and nominal peak concentrations of the fungicides used in the mixtures comprising three and four different compounds. Base concentrations were at 10% of the peak concentrations.

Fungicide	EC_{50} [µg/L]	Reference species[a]	Source	Concentration [µg/L]; 4 pesticides[d]	Concentration [µg/L]; 3 pesticides
Metalaxyl	743	*Pseudokirchneriella subcapitata*[b]	2	18.6	24.8
Prothioconazole	126	Several hyphomycete species[c]	3	3.2	4.2
Prochloraz	8	Several hyphomycete species[c]	3	0.2	-
Pyrimethanil	1200	*Pseudokirchneriella subcapitata*[b]	4	30.0	40.0

[a]If available, hyphomycete species were chosen as reference organisms, if not available we used *Pseudokirchneriella subcapitata*.

[b]EC_{50} values for *Pseudokirchneriella subcapitata* are based on 72-h acute toxicity tests after OECD 201[5].

[c]Data calculated from mean EC_{50} values of several hyphomycete species of pesticides from same substance group as the used ones.

[d]The pesticide mixture with four pesticides consisted of metalaxyl, prothioconazole, pyrimethanil, and prochloraz, while in the mixture with three pesticides prochloraz was discarded due to technical difficulties in some regions and during some cycles.

Supplementary Table 2: Percentage change in the decomposed leaf mass between the fungicide treatment and respective controls (%; with 95% confidence intervals), sample sizes, as well as the t-test results separated by biogeographical regions and cycles. Bold p-values indicate statistical significance.

Region	Cycle	Percentage change	95% confidence interval	n	df	t-ratio	p-value
Denmark	1	-23.1	-37.0 to -9.1	7	36	-2.4	**0.023**
	2	-37.6	-55.3 to -19.9	7	36	-3.1	**0.004**
	3	0.0	-40.0 to 21.6	7	36	-0.4	0.671
Germany	1	-53.1	-72.1 to -34.1	7	36	-4.0	**0.003**
	2	-19.3	-36.3 to -2.2	7	36	-1.6	0.114
	3	10.0	-7.0 to 26.9	7	36	0.8	0.404
Sweden	1	-18.6	-30.6 to -6.6	6	30	-2.2	**0.033**
	2	-15.6	-31.8 to 0.6	6	30	-1.4	0.174
	3	-1.4	-19.1 to 16.3	6	30	-0.1	0.908

Supplementary Table 3: Influence of explanatory variables on the sporulation of cosmopolitan (occurring in all analysed biogeographical regions) and non-cosmopolitan (occurring in two biogeographical regions) aquatic hyphomycete taxa tested by type II ANOVAs, separated by biogeographical region and explanatory variables. Only statistical significant explanatory variables are shown.

	Hyphomycete	Region	Explanatory variable	df	F	p-value
Cosmopolite taxa	*Articulospora tetracladia*	Denmark	Fungicide × Cycle	2	15.2	**< 0.001**
		Germany			NS[a]	
		Sweden	Cycle	2	8.2	**0.002**
	Flagellospora curvula	Denmark			NS	
		Germany			NS	
		Sweden			NS	
	Tetrachaetum elegans	Denmark			NS	
		Germany	Cycle	1	11.2	**0.004**
		Sweden			LS[b]	
Non-cosmopolite taxa	*Alatospora sp.*	Denmark			NF[c]	
		Germany			LS	
		Sweden	Fungicide	1	14.0	**< 0.001**
	Anguillospora sp	Denmark			LS	
		Germany			NF	
		Sweden	Cycle	2	10.8	**< 0.001**
	Mycocentrospora clavata	Denmark			NS	
		Germany			NF	
		Sweden			LS	
	Tetracladium sp.	Denmark	Cycle	2	13.8	**< 0.001**
		Germany			NS	
		Sweden			NF	
	Tetracladium marchalianum	Denmark	Cycle	2	6.5	**0.004**
		Germany			NS	
		Sweden			NF	

[a]NS: no significant explanatory variables

[b]LS: sporulation too low to build a stable model

[c]NF: not found

Additional experiment in the South Eastern Highlands (Australia)

The colonisation and decomposition cycles 2 and 3 showed a non-significant decrease in leaf decomposition in the fungicide treatment compared to the respective controls ($p \geq 0.081$; Supplementary Fig. 1). However, and in accordance with the experiments in the other regions, the differences between control and fungicide treatment decreases from cycle 2 to 3 (Supplementary Table 4).

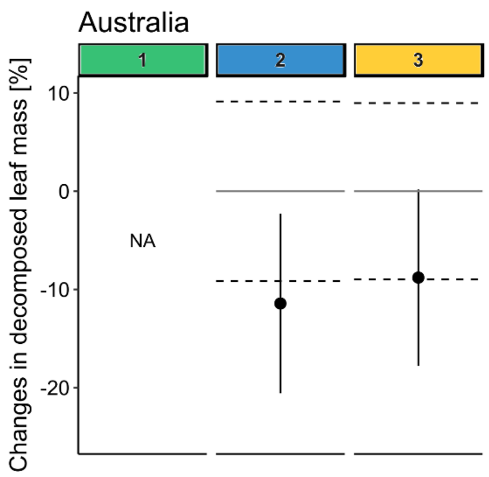

Supplementary Fig. 1: Percentage change in the decomposed leaf mass between the fungicide treatment and the respective controls (%; with 95% confidence intervals, solid horizontal lines represent controls, dashed lines indicate corresponding 95% confidence intervals) for the different cycles (numbers on top; n = 7) from the Australian experiment.

Supplementary Table 4: Percentage change in the decomposed leaf mass between the fungicide treatment and respective controls (%; with 95% confidence intervals), sample sizes, as well as the type II ANOVA results from the Australian experiment.

Region	Cycle	Percentage change	95% confidence interval	n	df	t-ratio	p-value
Australia	2	-11.4	-20.6 to -2.2	7	24	-1.8	0.081
	3	-8.8	-17.8 to -0.2	7	24	-1.4	0.166

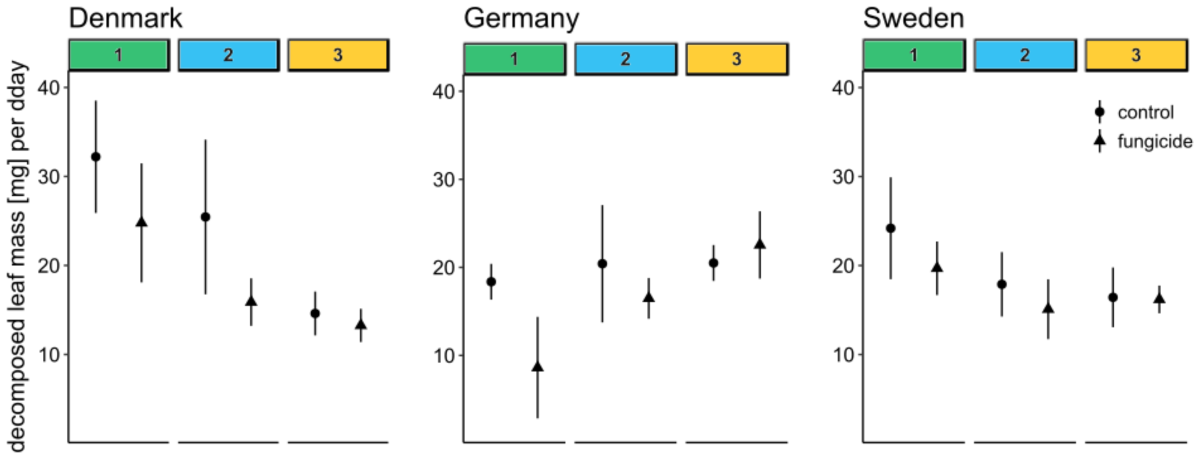

Supplementary Fig. 2: Mean decomposed leaf mass [mg] (with 95% confidence intervals) per degree day (dday) for the different cycles (numbers on top) and treatments (circle: control; triangle: fungicide treatment).

Supplementary Table 5: Influence of explanatory variables on the aquatic hyphomycete taxa richness tested by type III ANOVAs, separated by biogeographical regions and explanatory variables. Bold p-values indicate statistical significance.

Region	Explanatory variable	df	LRT	p-value
Denmark	Fungicide	1	0.0	1.00
	Cycle	2	34.7	**< 0.001**
	Fungicide × Cycle	2	4.1	0.126
Germany	Fungicide	1	1.8	0.184
	Cycle	1	0.5	0.481
	Fungicide × Cycle	1	0.0	0.879
Sweden	Fungicide	1	2.8	0.093
	Cycle	2	13.2	**0.001**
	Fungicide × Cycle	2	1.9	0.387

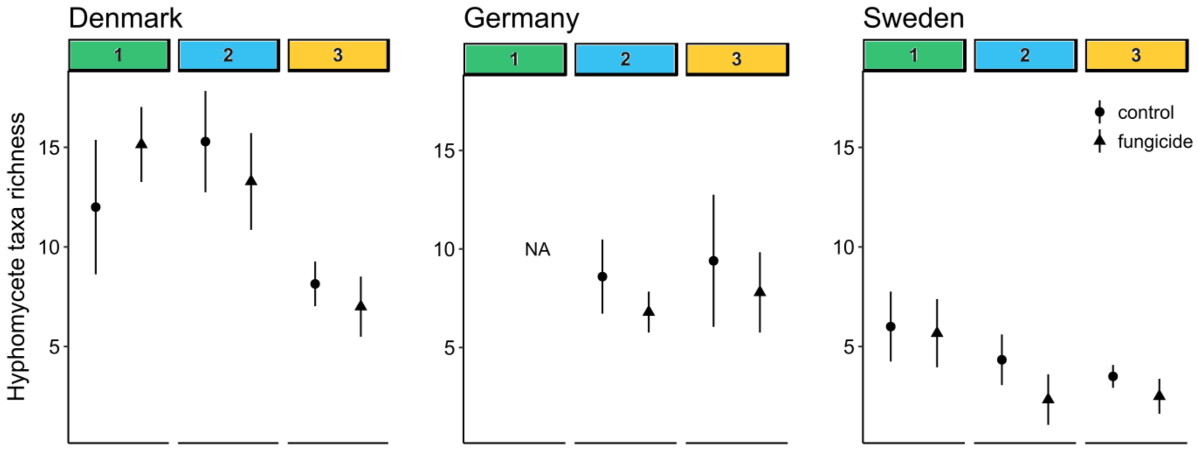

Supplementary Fig. 3: Mean hyphomycete taxa richness (with 95% confidence intervals) for the different cycles (numbers on top) and treatments (circle: control; triangle: fungicide treatment)

Fungicide analysis

Water samples were collected from four randomly chosen replicates per treatment at different time points during peak and base exposures (for time points, see Supplementary Table 7) and concentrated using solid phase extraction (SPE; HLB 3 cc 60 mg extraction cartridges). SPE columns were conditioned with 5 mL acetone and 5 mL acetonitrile (both HPLC grade) and equilibrated with 5 mL ultrapure water. An aliquot of 0.1 L water was loaded into the SPE column at a velocity of 3 - 4 mL/min. The columns were dried under a nitrogen flow for 30 min and stored at -20°C until analysis. Before elution, the columns were dried accordingly and eluted with 4 mL acetonitrile followed by 4 mL acetone (both liquid chromatography grade). The eluate was evaporated to dryness under a gentle nitrogen flow at room temperature and reconstituted in 0.5 mL methanol:water (1:1, v/v, methanol liquid chromatography grade, ultrapure water). Finally, the extract was centrifuged (3000 rpm, 7 minutes), and the supernatant was used for further analysis. Depending on the nominal concentrations, samples were 5- or 50-fold diluted with methanol:water (1:1, v/v) to fit within the linear range of the calibration curve.

The samples and standards were analysed using a liquid chromatography high-resolution mass-spectrometry (LC-HRMS) Orbitrap system according to Fernández et al.[6]. Quantitative analyses were performed by interpolating the data according to the respective matrix-matched calibration curve. All studied fungicides showed a linear range from 0.5 to 200 µg/L in pure solvents (methanol:water) as well as in the different matrices studied. Additionally, the matrix effect was calculated via post-extraction spike using pre-filtered stream water from each biogeographical region. The effect of the matrix on the performance of the method differed between the stream water samples from each biogeographical region, and it was affected also by the sample dilution factor (5- and 50-fold dilution). Consequently, different limits of quantification were obtained (LOQs; Supplementary Table 6). Because prothioconazole has a high degradation rate[4] and was applied at relatively low nominal concentrations during this study, the pesticide was not detectable in the samples based on the applied methods.

Supplementary Table 6: Limits of quantifications (LOQs) of the individual fungicides (Chemical Abstract Service, CAS, given in brackets) in ultrapure water and stream water that was used as test medium in the individual experiments from the different geographical regions (Denmark, Germany, and Sweden). The LOQ values represent the lowest calibration limit in each matrix, adjusted to the extraction method considering a sample volume of 0.1 L.

		Metalaxyl (57837-19-1)	Prochloraz (67747-09-5)	Pyrimethanil (53112-28-0)
Region	Dilution step	LOQ [µg/L]	LOQ [µg/L]	LOQ [µg/L]
-	Ultrapure water	0.005	0.0025	0.0025
Denmark & Germany	Undiluted	0.025	0.050	0.025
	1:5 dilution	0.005	0.050	0.025
	1:50 dilution	0.005	0.0025	0.005
Sweden	Undiluted	0.050	0.025	0.025
	1:5 dilution	0.025	0.005	0.025
	1:50 dilution	0.005	0.005	0.025

Supplementary Table 7: Mean measured pesticide concentrations (with standard deviations, from four randomly chosen replicates), separated by biogeographical regions and sampled time points during the peak and the base exposure. Nominal initial peak concentrations in the mixtures containing three or four fungicides were: metalaxyl 24.8 and 18.6 µg/L, prothioconazole 4.2 and 3.2 µg/L, prochloraz 0.2 µg/L (only in the mixture containing four fungicides), and pyrimethanil 40.0 and 30.0 µg/L. Note that prothioconazole was not detectable due to its high degradation rate and therefore is not reported.

Region	Exposure	Hours after peak application	Metalaxyl [µg/L]	Prochloraz [µg/L]	Pyrimethanil [µg/L]
Denmark	peak	0	22.1 ± 2.0	0.10 ± 0.02	30.5 ± 2.6
	base	50	2.3 ± 0.4	0.008 ± 0.005	3.9 ± 0.6
Germany	peak[a]	0	19.5 ± 8.1	0.08 ± 0.01	34.1 ± 6.0
	peak[b]	0	29.0 ± 7.0	NA	49.9 ± 10.3
	peak	24	32.3 ± 7.3	NA	41.9 ± 7.9
	peak	48	27.3 ± 3.3	NA	31.5 ± 3.9
	base	50	1.7 ± 0.3	NA	2.7 ± 0.6
	base	86	1.6 ± 0.2	NA	2.4 ± 0.5
	base	122	2.8 ± 0.4	NA	2.2 ± 0.2
	base	242	0.09 ± 0.01	NA	1.2 ± 0.5
Sweden	peak	0	28.0 ± 10.4	NA	35.7 ± 10.4
	peak	24	24.9 ± 10.5	NA	20.1 ± 5.7
	peak	48	21.9 ± 6.2	NA	21.4 ± 4.5
	base	50	1.2 ± 0.3	NA	3.3 ± 0.6
	base	86	0.9 ± 0.2	NA	2.1 ± 0.4
	base	122	0.8 ± 0.1	NA	1.9 ± 0.3

[a]The fungicide mixtures of the peak exposure of cycle 1 in Germany included prochloraz, which was excluded in later cycles due to technical difficulties.

[b]The fungicide mixtures of the peak exposures of cycles 2 and 3 in Germany consisted only of the fungicides prothioconazole, pyrimethanil and metalaxyl.

NA = not applied.

All fungicides were below the level of detection in all control samples and thus are not listed in Supplementary Table 7.

Water quality parameters

The average water temperature was recorded every 30 min during microbial colonisation of the leaf material in streams and in each control of the randomly distributed microcosms during the experiment to calculate the decomposed leaf mass per degree day (Supplementary Table 8).

The ion contents in Germany were measured using a field photometer PF-12 as well as related VISOCOLOR ECO kits, and they were measured in Sweden using an automated photometric analyser. In Denmark, the biological oxygen demand (BOD5) and concentrations of ammonia-N and ortho-phosphate were measured according to their European Standards (DS/EN 1899 1999, DS 11732 2005, and DS/EN 1189 1999, respectively). Nitrate-N was analysed using the Lachat-method (Lachat Instruments, USA, QuickChem. No. 10-107-06-33-A, salicylate method).

Supplementary Table 8: Abiotic water parameters. Temperature and pH values are given during colonisation of the leaf material in the field and the experimental part of the experiment, while water quality parameters were measured directly after water exchanges.

		Australia	Denmark	Germany	Sweden
During colonisation	Temperature [°C]	18	9	8	9
	pH	7.2	7.4	7.4	6.6
During experiment	Temperature [°C]	18	15	18	11
	pH	NM	7.1	7.1	7.1
	Conductivity [µS/cm]	55	NM	84	455
	DOC [mg/L]	NM	1.3	< 1	48
	NH4-N [mg/L]	NM	0.026	< 0.1	0.023
	NO2-N [mg/L]	NM	NM	< 0.01	
	PO4-P [mg/L]	NM	0.01	< 0.2	0.013
	NO3-N [mg/L]	NM	0.54	< 0.1	
	Sum NO2+NO3 [µg/l]	NM	NM	NM	11
	BOD5 [mg/L]	NM	0.84	NM	NM

NM: Not measured.

Supplementary Table 9: Coordinates and times for leaf collection in the different biogeographical regions.

Region	Time	Latitude	Longitude
Australia	February 2015	34°55'20"S	149°10'49"E
Denmark	Autumn 2014	56°00'16"N	10°07'26"E
Germany	October 2013	49°12'07"N	8°08'37"E
Sweden[a]	Autumn 2014	49°12'39"N	8°13'15"E

[a]Leaves were imported from Landau, Germany.

Supplementary Table 10: Coordinates of colonisation of leaves from cycle 1.

Region	Name of stream	Latitude	Longitude
Australia	Cotter River	35°24'14"S	148°51'23"E
Denmark	Hulbaek	56°00'16"N	10°07'26"E
Germany	Sulzbach	49°15'43"N	7°57'36"E
Sweden	Pinglaström	59°46'19"N	17°45'19"E

Supplementary References

1. Fungicide Resistance Action Committee. Mode of action of fungicides— FRAC classification on mode of action 2017. (2017). Available at: http://www.frac.info/docs/default-source/publications/frac-mode-ofaction- poster/frac-moa-poster-march-2017f19b282c512362eb9a1eff 00004acf5d.pdf?sfvrsn=5fb84a9a_2.
2. EPA. The ECOTOXicology knowledgebase (ECOTOX). (2014). Available at: http://cfpub.epa.gov/ecotox/.
3. Dijksterhuis, J., van Doorn, T., Samson, R. & Postma, J. Effects of Seven Fungicides on Non-Target Aquatic Fungi. *Water. Air. Soil Pollut.* **222,** 421–425 (2011).
4. Lewis, K. A., Tzilivakis, J., Warner, D. J. & Green, A. An international database for pesticide risk assessments and management. *Hum. Ecol. Risk Assess. Int. J.* **22,** 1050–1064 (2016).
5. OECD. *Test No. 201: Freshwater Alga and Cyanobacteria, Growth Inhibition Test*. (Organisation for Economic Co-operation and Development, 2011).
6. Fernández, D. *et al.* Does nutrient enrichment compensate fungicide effects on litter decomposition and decomposer communities in streams? *Aquat. Toxicol.* **174,** 169–178 (2016).
